# Supplementary material for: Assessment of Diagnosis, Prognosis and Immune Infiltration Response to the Expression of the Ferroptosis-Related Molecule HAMP in Clear Cell Renal Cell Carcinoma
Source: Int J Environ Res Public Health. 2023 Jan 4;20(2):913. doi: 10.3390/ijerph20020913 (PMC9858726; doi:10.3390/ijerph20020913)
Supplement: Supplementary file 1 [file ijerph-20-00913-s001.zip › ijerph-2056076-supplementary.pdf]

Supplementary Material

Table S1. (A) Association with overall survival and clinicopathologic characteristic in TCGA patients using Cox regression. (B) Multivariate survival using Cox regression.

Clinical Characteristics	HR (95% CI)	*p*-Value
A		
Age	1.77 (1.30–2.40)	<0.001
T stage	2.92 (2.10–4.06)	<0.001
N Stage	3.45 (1.83–6.51)	<0.001
M stage	4.39 (3.21–6.00)	<0.001
HAMP	1.83 (1.34–2.49)	<0.001
B		
T Stage	1.49 (0.88–2.51)	0.136
N Stage	2.16 (1.11–4.20)	0.023
M Stage	3.31 (2.04–5.36)	<0.001
HAMP	1.37 (0.86–2.60)	0.182

Table S2. The intersection genes selected by GEPIA and R.

Only GEPIA	Only R	GEPIA AND R
ZPLD1	PTPRN2-AS1	ZNF385A
ZBED2	CRYBB1	VSIG4
TRNP1	LIMK1	VAV1
TLR8	GNA15	UCP2
ST3GAL5	BASP1	TYROBP
SNORA70	PYCARD	TREM2
SIGLEC10	JPT1	TNFSF13B
SELPLG	AC011899.2	TNFAIP8L2
SCIMP	HTRA4	STAC3
SAMSN1	LINC02513	SPI1
RP4-736H5.3	IGFLR1	SNX20
RP11-9M16.3	TRPM2	SLAMF8
RP11-83C7.1	LY96	SIGLEC9
RP11-742B18.1	PLA2G2D	RNASE2
RP11-556E13.1	CSTA	RGS10
RP11-153M7.1	RARRES1	RAC2
RP1-111D6.3	APOC1	PCED1B-AS1
RNASE3	BATF	OSCAR
PTPRO	NCF4	MARCO
PLEK	PLTP	LILRB4
PLB1	CD72	LAT2
NRSN1	CARD11	LAPTM5
MCEMP1	LINC01857	LAIR1
LINC01111	C1R	IKBKE
LINC01109	DOK3	GPR84
LINC01094	PLAUR	GAL3ST4
LHFPL2	LY86	FCGR2B
LGALS9	WAS	FCGR1B
ITGB2	FERMT3	FCGR1A
IL2RA	CEBPA	FCER1G
IL21R	SH3BP1	ERC2
IGF2BP3	FCGR1CP	CD14

HGF	APOC2	C1QC
HCK	POU2F2	C1QB
FPR1	DNAJC5B	C1QA
FCGR3A	CTSZ	BCL2A1
FCGR1C	MILR1	ARPC1B
EBI3	KCNN4	AIF1
DLEU7	APOE	AC092484.1
DCBLD2	CD37	
CYBB	FZD2	
CSF1R	MOCOS	
CRB2	NOD2	
CR1	HCST	
CLEC6A	TMEM150B	
CD86	SLC29A3	
CD80	C1S	
CD53	REEP4	
CD4	SAA1	
CD163	LPAR5	
CCR1	PI3	
CASP5	GPX1	
C3AR1	RGS19	
ATP5G2P4	NCF1	
ARL4C	S100A11	
ALOX5AP	GFPT2	
ADORA3	FTL	
ADGRE1	FCHO1	
ADAMDEC1	AC093895.1	
AC023590.1	ADAM8	
AC011899.9	NCF1C	

Table S3. Gene sets enriched in *HAMP* high expression phenotype.

Gene Set Name	NES	NOM p-val	FDR q-val
KEGG_OXIDATIVE_PHOSPHORYLATION	2.086371	0	0.031508
KEGG_PARKINSONS_DISEASE	2.00546	0.019493	0.045734
GO_CYTOKINE_ACTIVITY	2.535268	0	3.66×10^{-04}
GO_IMMUNE_RECEPTOR_ACTIVITY	2.439605	0	7.67×10^{-04}
GO_T_CELL_PROLIFERATION	2.330577	0	3.76×10^{-05}
GO_T_CELL_ACTIVATION_INVOLVED_IN_ IMMUNE_RESPONSE	2.258216	0	1.87×10^{-04}

Table S4. Gene sets enriched in *HAMP* low expression phenotype.

Gene Set Name	NES	NOM p-val	FDR q-val
KEGG_B_CELL_RECEPTOR_SIGNALING_PATHWAY	−2.02156	0.003984	0.010226
KEGG_JAK_STAT_SIGNALING_PATHWAY	−2.2941	0	9.30×10^{-04}
KEGG_SMALL_CELL_LUNG_CANCER	−1.78866	0.023857	0.046139
KEGG_PANCREATIC_CANCER	−1.88035	0.01232	0.027684
KEGG_AUTOIMMUNE_THYROID_DISEASE	−1.92458	0.01378	0.020369
KEGG_NON_SMALL_CELL_LUNG_CANCER	−2.02375	0.002033	0.010281
GO_AMMONIUM_ION_METABOLIC_PROCESS	−2.50828	0	0.006186
GO_BETA_CATENIN_BINDING	−2.3993	0	0.027118
